# Initial Experience of a Community Gastroenterology Practice with Ultraslim Colonoscopy

**DOI:** 10.7759/cureus.4663

**Published:** 2019-05-14

**Authors:** Rajesh Essrani, Patrick Hickey, Hiral Shah

**Affiliations:** 1 General Internal Medicine, Geisinger Medical Center, Danville, USA; 2 Gastroenterology, Lehigh Valley Health Network, Allentown, USA

**Keywords:** ultrathin colonoscope, colonoscopy, colon polyp, colorectal cancer

## Abstract

Colonoscopies are performed for cancer screening as well as for other diagnostic and therapeutic reasons. It is considered successful if cecal intubation is achieved and adequate mucosa is visualized. It is not always possible to achieve cecal intubation due to multiple reasons such as sharp angulation or fixed segments of the colon and stricture. A pediatric colonoscope (PDC) and an ultrathin colonoscope (UTC) are used as a tool to negotiate sharp angulation and the fixed segments of the colon because their small diameter allows easy movement. An ultrathin colonoscope is used at many practices as a rescue in situations where standard colonoscopes have failed. Our study highlights the use of an ultraslim scope in both rescue situations and as the initial colonoscope of choice in an ambulatory endoscopy center.

## Introduction

The PCF-PH190L/I is among the new endoscopes Olympus has recently introduced as part of their EVIS EXERA III system (Olympus America Inc., PA, US). The ultraslim colonoscope has an outer diameter of 9.7 mm and a 3.2 mm working channel width. Its advantages include reduced physician fatigue and patient discomfort [1]. Cecal intubation rates are high with low mean cecal intubation times. It has enhanced the optical system with higher brightness, viewing angle, and ultra-high definition imaging quality. It has a responsive insertion technology (RIT), with passive bending, which allows more natural movement through acute bends in the colon, improving operator control for both pushing and twisting maneuvers. It can be used in patients with previous incomplete colonoscopies with less or no sedation. The purpose of this study is to review a series of patients who underwent colonoscopy with a newly acquired ultraslim colonoscope (Olympus PCF-PH190L/I) in a community ambulatory endoscopy center.

## Materials and methods

 A total of 13 colonoscopies were performed on 13 patients (10 women and three men), with a mean age of 64.5 years, by five different providers. Common indications for colonoscopy were screening for colorectal cancer (38.4%), a history of colon polyps (38.4%), changes in bowel habits (7.6%), abnormal computed tomography (CT) scan of the abdomen (7.6%), and heme-positive stool (7.6%).

## Results

The most common reason for the use of an ultraslim colonoscope was patient comfort (84.6 %), fixed sigmoid, preventing the passage of a pediatric colonoscope (7.7%), and prior failed colon (7.7%). Results showed diverticulosis (76.9%), internal hemorrhoids (46.1%), polyps (30.7%), rectal gastrointestinal stromal tumor (7.6%), and only one normal colonoscopy (7.6%) (Table 1). Cecal intubation was obtained in 92.3% of patients.

**Table 1:** Summation of study Table shows the number of patients who underwent ultrathin colonoscope (UTC), the number of providers who performed a colonoscopy, reasons for UTC, and findings. UTC: ultrathin colonoscope

Patient #	Gender	Age	Provider	Cecum Reached	Prior Failed Colon	Indication Stated? (If so what)	Adenoma	Reason for Ultra-Thin	Findings
1	Female	62	1	No	No	Change in bowel habits, weight loss	No	Fixed sigmoid preventing passage of the pediatric colonoscope	Possible rectosigmoid junction tumor ingrowth with polypoid lesion (lymphoid aggregates, no neoplasia/dysplasia), diverticulosis, internal hemorrhoids
2	Female	66	2	Yes	Yes	Abnormal CT scan	No	Prior failed colon	Normal colonoscopy
3	Male	76	3	Yes	No	Heme-positive stools	Yes	Patient comfort	Pedunculated polyp, necrotic-appearing area (biopsy with granulation tissue)
4	Female	69	2	Yes	No	Screening for colorectal cancer	No	Patient comfort	Severe sigmoid diverticulosis, internal hemorrhoids
5	Female	62	2	Yes	No	History of colon polyps	No	Patient comfort	Tortuous sigmoid colon, sigmoid diverticulosis
6	Female	69	4	Yes	No	History of colon polyps	Yes	Patient comfort	Rectal gastrointestinal stromal tumor, sigmoid polyp, sigmoid diverticulosis
7	Female	64	4	Yes	No	Screening for colorectal cancer	No	Patient comfort	Diverticulosis
8	Male	62	4	Yes	No	Screening for colorectal cancer	No	Patient comfort	Sigmoid diverticulosis, internal/external hemorrhoids
9	Female	56	2	Yes	No	History of colon polyps	Yes	Patient comfort	Ascending colon sessile serrated adenoma, diverticulosis, internal hemorrhoids
10	Male	74	3	Yes	No	Screening for colorectal cancer	No	Patient comfort	Severe diverticulosis
11	Female	65	2	Yes	No	History of colon polyps	No	Patient comfort	Severe diverticulosis, internal hemorrhoids
12	Female	51	2	Yes	No	Screening for colorectal cancer	Yes	Patient comfort	Cecal polyp, internal hemorrhoids
13	Female	63	5	Yes	No	History of colon polyps	No	Patient comfort	Severe diverticulosis

## Discussion

Colonoscopy is performed for both diagnostic and therapeutic reasons [2]. Diagnostic reasons include surveillance or screening for colon cancer [3], evaluating signs and symptoms suggestive of possible distal small bowel or colonic disease like gastrointestinal bleeding, evaluating response to therapy in patients with a known colonic disease (e.g., inflammatory bowel disease) [4-7], and evaluating pathologies found on imaging studies. Therapeutic indications include stricture dilation [8], stent placement [9-12], foreign body removal [13], colonic decompression, and treating bleeding lesions [14].

Colonoscopy is considered successful if cecal intubation is achieved and adequate mucosa is visualized. Preparation for colonoscopy typically involves the ingestion of a low-residue diet or clear liquids for at least one day before the examination, combined with an oral gastrointestinal lavage.

It is not always possible to achieve cecal intubation due to multiple reasons, such as sharp angulation or fixed segments of colon, stricture, diverticular disease, incomplete bowel preparation, diverticular disease, poor quality of bowel preparation, patient’s comorbidities, and patient non-compliance during the procedure, leading to repeat procedure; patient’s dissatisfaction, higher cost burden, and more time needed to diagnose the disease.

The main advantage of a small-caliber endoscope over a standard colonoscope is its greater flexibility and smaller diameter, making it feasible for patients with narrowed colons [15].

Pediatric colonoscopes (PDC) and ultrathin colonoscopes (UTC) are used as a tool to negotiate sharp angulation and fixed segments of the colon because their small diameter allows easy movement. Daiki Nemoto et al. performed a randomized, prospective controlled trial to compare UTC (diameter 7.0 mm) with PDC to compare the degree of pain and cecal intubation rate. There was a major difference in the reported pain. UTC was tolerated better as compared to the pediatric scope, and cecal intubation rates were 97.4% with UTC and 92.1% with PDC [16].

An ultrathin colonoscope comes in different diameters, with a greater imaging quality and bending capability to increase the cecal intubation rate and intubation time and decrease physician/patient discomfort level. Luo et al. formed a prospective randomized controlled trial comparing ultrathin caliber colonoscopy (diameter 9.2 mm) with the standard colonoscope (SC) to compare first and rescue successful cecal intubation rates, subject satisfaction scores, and sedation requirements. The study signifies that there was no statistically significant difference in the first successful cecal intubation rate between the UTC and SC groups, less sedation was used in UTC patients, and a similar satisfaction score was found [17].

UTC has performance characteristics like those of the SC; however, it has the advantage that subjects feel less pain so the most common indication is the patient’s comfort. The mechanism of pain reduction with UTC is not known, although it is likely that the smaller caliber and flexible instrument places less tension on the colonic wall and, with its flexibility, may even conform to the colonic anatomy rather than form loops. There are several drawbacks with UTC. The extremely thin insertion tube is highly flexible and can be difficult to advance, particularly in the proximal colon [17-18]. A narrower diameter instrumental channel makes the suction of stool/debris more difficult and limits the size of therapeutic accessories that can be used. Therefore, UTC is suitable for a screening indication but is less suitable for complicated therapeutic endoscopies.

Our study showed that the use of an ultraslim colonoscope had a cecal intubation rate of 92.3%. Patients with a prior failed colonoscopy had successful cecal intubation. A large number of patients were selected to have a colonoscopy with an ultraslim scope for patient and provider comfort. Patients reportedly prefer ultraslim colonoscopy for comfort and reduced sedation requirements, making it an ideal choice for community gastroenterologists [19-20].

This article has earlier been presented as a poster in ACG 2017 (Essrani E, Hickey P, Shah H, Blanco P. Initial experience of a community gastroenterology practice with ultra-slim colonoscopy: a case series; ACG 2017).

## Conclusions

UTC is used at many practices as a rescue in situations where standard colonoscopes have failed. Our study highlights the use of an ultraslim scope in both rescue situations and as the initial colonoscope of choice in an ambulatory endoscopy center. Further studies are needed to evaluate the use of ultraslim colonoscopy as the first choice and its indications and advantages.

## References

[1] Garborg KK, Loberg M, Matre J, Holme O, Kalager M, Hoff G, Bretthauer M (2012). Reduced pain during screening colonoscopy with an ultrathin colonoscope: a randomized controlled trial. Endoscopy.

[2] Rex DK, Schoenfeld PS, Cohen J (2015). Quality indicators for colonoscopy. Gastrointest Endosc.

[3] Canadian Task Force on Preventive Health C (2016). Recommendations on screening for colorectal cancer in primary care. CMAJ.

[4] Pera A, Bellando P, Caldera D (1987). Colonoscopy in inflammatory bowel disease. Diagnostic accuracy and proposal of an endoscopic score. Gastroenterology.

[5] Lux G, Fruhmorgen P, Phillip J, Zeus J (1978). Diagnosis of inflammatory diseases of the colon: comparative endoscopic and roentgenological examinations. Endoscopy.

[6] Waye JD (1977). The role of colonoscopy in the differential diagnosis of inflammatory bowel disease. Gastrointest Endosc.

[7] Leighton JA, Shen B, Baron TH (2006). ASGE guideline: endoscopy in the diagnosis and treatment of inflammatory bowel disease. Gastrointest Endosc.

[8] Bannura GC, Cumsille MA, Barrera AE, Contreras JP, Melo CL, Soto DC (2004). Predictive factors of stenosis after stapled colorectal anastomosis: prospective analysis of 179 consecutive patients. World J Surg.

[9] van Hooft JE, van Halsema EE, Vanbiervliet G (2014). Self-expandable metal stents for obstructing colonic and extracolonic cancer: European Society of Gastrointestinal Endoscopy (ESGE) Clinical Guideline. Endoscopy.

[10] Baron TH, Wong Kee Song LM, Repici A (2012). Role of self-expandable stents for patients with colon cancer (with videos). Gastrointest Endosc.

[11] Kim JS, Lee KM, Kim SW (2014). Preoperative colonoscopy through the colonic stent in patients with colorectal cancer obstruction. World J Gastroenterol.

[12] Pothuri B, Guirguis A, Gerdes H, Barakat RR, Chi DS (2004). The use of colorectal stents for palliation of large-bowel obstruction due to recurrent gynecologic cancer. Gynecol Oncol.

[13] Lake JP, Essani R, Petrone P, Kaiser AM, Asensio J, Beart RW Jr (2004). Management of retained colorectal foreign bodies: predictors of operative intervention. Dis Colon Rectum.

[14] Jensen DM, Machicado GA (1988). Diagnosis and treatment of severe hematochezia. The role of urgent colonoscopy after purge. Gastroenterology.

[15] Sato K, Shigiyama F, Ito S, Kitagawa T, Tominaga K, Suzuki T, Maetani I (2013). Colonoscopy using a small-caliber colonoscope with passive-bending after incomplete colonoscopy due to sharp angulation or pain. Surg Endosc.

[16] Nemoto D, Utano K, Endo S, Isohata N, Hewett DG, Togashi K (2017). Ultrathin versus pediatric instruments for colonoscopy in older female patients: a randomized trial. Dig Endosc.

[17] Luo DJ, Hui AJ, Yan KK (2012). A randomized comparison of ultrathin and standard colonoscope in cecal intubation rate and patient tolerance. Gastrointest Endosc.

[18] Yamauchi H, Kida M, Okuwaki K (2015). Passive-bending, short-type single-balloon enteroscope for endoscopic retrograde cholangiopancreatography in Roux-en-Y anastomosis patients. World J Gastroenterol.

[19] Sofi AA, Nawras A, Khan MA, Howden CW, Lee WM (2017). Meta-analysis of the performance of ultrathin vs. standard colonoscopes. Endoscopy.

[20] Tox U, Schumacher B, Toermer T (2013). Propofol sedation for colonoscopy with a new ultrathin or a standard endoscope: a prospective randomized controlled study. Endoscopy.

